# A Novel Assay Reveals Hygrotactic Behavior in *Drosophila*

**DOI:** 10.1371/journal.pone.0119162

**Published:** 2015-03-04

**Authors:** Feiteng Ji, Yan Zhu

**Affiliations:** 1 State Key Laboratory of Brain and Cognitive Science, Institute of Biophysics, Chinese Academy of Sciences, Beijing, China; 2 University of the Chinese Academy of Sciences, Beijing, China; Alexander Fleming Biomedical Sciences Research Center, GREECE

## Abstract

Humidity is one of the most important factors that determines the geographical distribution and survival of terrestrial animals. The ability to detect variation in humidity is conserved across many species. Here, we established a novel behavioral assay that revealed the thirsty *Drosophila* exhibits strong hygrotactic behavior, and it can locate water by detecting humidity gradient. In addition, exposure to high levels of moisture was sufficient to elicit proboscis extension reflex behavior in thirsty flies. Furthermore, we found that the third antennal segment was necessary for hygrotactic behavior in thirsty flies, while arista was required for the avoidance of moist air in hydrated flies. These results indicated that two types of hygroreceptor cells exist in *Drosophila*: one located in the third antennal segment that mediates hygrotactic behavior in thirst status, and the other located in arista which is responsible for the aversive behavior toward moist air in hydration status. Using a neural silencing screen, we demonstrated that synaptic output from the mushroom body α/β surface and posterior neurons was required for both hygrotactic behavior and moisture-aversive behavior.

## Introduction

Water is the most abundant and important nutrient for the animal body and the proper regulation of body water is absolutely essential for survival. Animals have evolved sensitive osmoreceptors to detect fluctuations in body water content. These receptors trigger feelings of thirst that drives animals to take in water [1–4]. To obtain sufficient water for survival, it is important for the animals to locate water sources in the environment. This is important especially for insects because their large surface-area-to-volume ratio makes them highly susceptible to dehydration. Previous studies demonstrated that insects possess two distinct systems to detect external water: the gustatory system for liquid water [5–7], and the hygrosensory system for water vapour in the air [8–18]. In *Drosophila*, PPK28, a member of the degenerin/epithelial sodium channel family, labels the gustatory water sensory neurons [6]. Mutation of the *ppk28* gene in flies eliminates both the cellular and behavioral responses to liquid water. However, the precise molecular mechanisms of hygrosensation in insects remain to be elucidated. Previous studies indicated the existence of specific hygroreceptors located in the insects’ antennae [10–19]. Two transient receptor potential channels—Nan and WTRW—were shown to play different roles in detecting dry or moist air respectively in *Drosophila* [15]. A recent study provided evidence that hygrosensation in *C*. *elegans* involves both mechanosensory and thermosensory pathways [20], suggesting a rather complicated process for hygrosensation.

The detection of external water through gustatory reception occurs only after the sensory neurons contact the water [5–7]. Thus, insects must possess other system(s) to identify water sources prior to their physical contact with water. The visual system of insects is probably inadequate to find water due to the limited spatial resolution of this system [21], especially under low-light conditions. Therefore, it is plausible to hypothesize that insects possess specific hygrosensory systems evolved for the detection of external water sources. So far, most studies on hygrosensation of insects were performed using a T-maze humidity choice assay, in which the insects were allowed to make a choice between dry air and moist air [14–15]. However, despite its successful application in mutant screens, the T-maze assay is not sufficient to test complex responses to moisture in thirsty animals, due to only two relative humidities provided in the assay.

Here, we designed a novel assay in which a water source was supplied in an open environment. Thus, the assay can display the water-seeking behavior of thirsty animals under natural conditions. Using this assay, we observed more behavioral details when thirsty flies seek water, including migration along the moisture gradient and proboscis extension reflex near the water source. In addition, we demonstrated that *Drosophila* had two separated hygrosensory pathways that mediated opposite behavioral responses to moisture, according to the body osmolality status. In the central nervous system, mushroom body α/β lobe might function as a high-order brain center for receiving and processing the humidity stimuli transmitted by both hygrosensory pathways.

## Materials and Methods

### Fly stocks

All fly stocks were maintained on standard fly medium at 25°C with a relative humidity of 60%. *NP3061-Gal4*, *NP5286-Gal4*, *17d-Gal4* and *UAS-TNT* were kindly provided by Aike Guo (Institute of Biophysics, CAS, Beijing, China). *Orco-Gal4*, *GH146-Gal4*, *elav-Gal4*, *nan* ^*36a*^, *UAS-wtrw* ^*RNAi1*.*15*^, *UAS-wtrw* ^*RNAi2*.*8*^ and *UAS-wtrw* ^*RNAi3.1[15]*^ were kindly provided by Yi Rao (Peking University, Beijing, China). *Canton-S* (*CS*), *UAS-mCD8*::*GFP*, *Orco* ^*1*^ and *Orco* ^*2*^ were obtained from the Bloomington *Drosophila* Stock Center.

### Hygrotaxis assay

The apparatus designed for hygrotactic studies was made of a culture dish (ϕ55 mm; height, 10 mm) covered with a 100 mesh nylon net. 50 flies aged between four and six days were placed inside the apparatus, and were then dehydrated in a chamber with anhydrous calcium chloride (25°C; relative humidity, ~10%) for the times indicated. The apparatus with dehydrated flies was then placed above a water-containing vial (ϕ10 mm). The distance between the nylon net and water surface was 1 mm. The locomotion of dehydrated flies was record by a digital camera. Hygrotactic behavior assays were performed at 25°C with a relative humidity of 40~50%.

To quantify the hygrotactic behavior, we defined a circular region (ϕ25 mm) in the center of the dish and counted the number of flies in the region during the test. The aggregation values at different time points during the test were calculated using the following formula: (NO_t_—NO_0_) / NO_sum_. Here, NO_t_ is the number of flies in the defined region at the moment; NO_0_ is the number of flies in the region at the beginning of test; the total number of flies (NO_sum_) in our tests was 50. The hygrotaxis index is defined as the average of all aggregation values measured at five-second intervals within each five-minute test.

To examine the behavioral responses towards other volatile compounds in dehydrated flies, water was replaced by selected volatile compound. Other experimental conditions were the same as described above.

Unless otherwise indicated, flies used in the hygrotaxis assays were female.

### Ablation experiments

Female flies aged four to six days were paralyzed by CO_2_ exposure. The sensory organs (arista, third antennal segment, maxillary palp and proboscis) were removed using fine forceps. After ablation, the flies were allowed to recover for 24 hours under normal culture condition. For the flies with proboscis removed, dehydration was performed one hour after ablation, as these flies are unable to eat or drink. All flies were dehydrated for eight hours (25°C; relative humidity, ~10%) before hygrotactic tests.

### Proboscis extension reflex (PER) assay

A single female fly aged four to six days was introduced into a hole (ϕ10 mm; height, 2 mm) in the perspex sheet covered with a 100 mesh nylon net. The distance between test holes in the perspex sheet is no less than 10 mm. The flies kept in the holes were dehydrated in a chamber with anhydrous calcium chloride (25°C; relative humidity, ~10%) for eight hours, then they were transferred to the assay environment (25°C; relative humidity, 40~50%). To examine the proboscis extension response of the flies, a water-saturated swab was put above the dehydrated flies, and the swab was kept about 1 mm away from the nylon net. The flies that fully extended their proboscis at least twice within 10 seconds were counted as responders. The percentage of PER was calculated as the number of responders divided by the total number of flies. 50 flies were tested in each trial, and eight trials were performed for each condition or genotype.

### T-maze humidity choice assay

T-maze humidity choice assays were performed as described previously [14–15]. The apparatus contains two tubes, one filled with moist air (~99% RH), and one with dry air (~3% RH). 50 female flies aged between four and six days were placed between the two tubes, and allowed to make a choice between the two types of air. After 5 minutes, the number of flies in each tube was counted, and the preference index (PI) for moist air was calculated using the following formula: PI = (flies’ number in moist air—flies’ number in dry air) / total number of flies in the test.

### Water-drinking behavior assay

Water-drinking behavior assays were carried out in the same apparatus used in the PER assays. After the flies were dehydrated for eight hours (25°C; relative humidity, ~10%), the nylon net was wetted with a water-saturated swab. The flies that showed immediate drinking behavior (within 2 seconds) after touching the water were counted as responders. The drinking rate was calculated as the number of responders divided by the number of tested flies (excluding the few flies that did not contact wet nylon net in the test). 50 female flies were tested in each trial, and eight trials were performed for each genotype.

### Measurement of weight loss and water intake

50 female flies at the age of four to six days were introduced into the dish used in the hygrotaxis assay, and the dish was weighed before and after the eight-hour dehydration. The loss of weight during dehydration equals to the difference of the dish weights before and after dehydration. After dehydration, 1 ml water was distributed on the nylon net and the dehydrated flies were fed with water for 10 minutes. Because flies tend to stay on the surface of the nylon net, almost flies with normal locomotor ability have access to the water. Then, the water was removed, and the dish with flies was weighed again. The water intake of flies within 10 minutes was measured as the difference of the weights of the dish before and after water-feeding.

### Locomotor ability test

A culture dish (ϕ55 mm) was used for locomotor ability tests. The culture dish was filled with 1% agar, leaving a thin space (~2 mm height) on the top for movement of flies. A single female fly aged four to six days was placed into the culture dish, and its locomotion was recorded using a digital camera. The locomotion distance within the first 10 seconds was measured using a program written in MATLAB (2012a, MathWorks). The flies that underwent ablation were allowed to recover for 24 hours before the test.

### Immunohistochemistry

Immunohistochemistry and confocal imaging were performed as described previously [22]. Rabbit anti-GFP antibody (Invitrogen, dilution 1:500) and mouse anti-nc82 antibody (DSHB, dilution 1:100) were used as primary antibodies. Goat anti-Rabbit antibody (Alexa Fluor 488-conjugated, Jackson ImmunoResearch, dilution 1:500) and Goat anti-Mouse antibody (TRITC-conjugated, Jackson ImmunoResearch, dilution 1:500) were used as secondary antibodies.

## Results

### Thirsty flies show hygrotactic behavior

**Fig 1:**
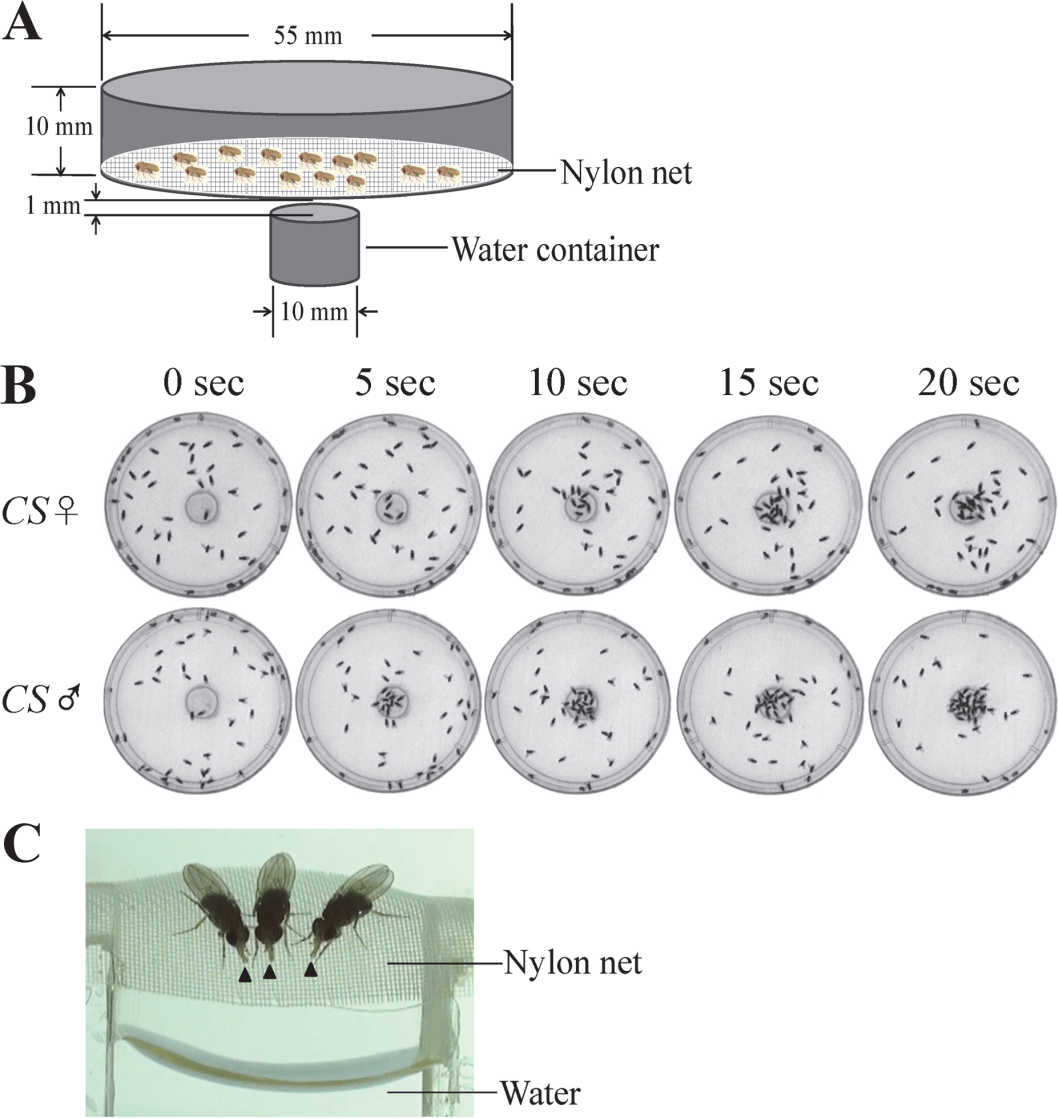
Hygrotactic behavior in thirsty *Drosophila*. (**A**) Schematic diagram of the apparatus used in the hygrotactic behavior assay. (**B**) Flies dehydrated for 8 hours migrated toward and aggregated near the water source rapidly. (**C**) PER behavior induced by moist air in dehydrated wild type flies (female; eight-hour dehydration). Arrowheads indicate extended proboscises.

We designed a novel assay to test the behavioral responses of thirsty flies to moisture. 50 flies were kept in a culture dish that was covered with a nylon net. After dehydration for 6 to 10 hours (25°C; relative humidity, ~10%), the dish was placed above a water-containing vial, leaving 1mm between the nylon net and the water surface (Fig. 1A). The dehydrated flies immediately migrated toward the water and aggregated above the water source (Fig. 1B). At the same time, the flies exhibited proboscis extension reflex (PER) and attempted to drink water even though they could not touch the water (Fig. 1C). The aggregation phenomenon continued for up to 20 minutes, after which the flies scattered gradually. The visual system is not necessary for the hygrotactic behavior, since this behavior was not diminished in darkness (data not shown). Additionally, we showed that the hygrotactic behavior was triggered by dehydration rather than starvation, since the wild type flies starved with water for 10 hours did not exhibit hygrotactic or PER behavior in the above assay (S1 Fig.), and feeding water to the thirsty flies for 10 minutes abolished these behaviors as well. Other volatile compounds, including ethanol, acetic acid and ethyl acetate, did not induce similar behaviors as water in thirsty flies (S2 Fig.).

**Fig 2:**
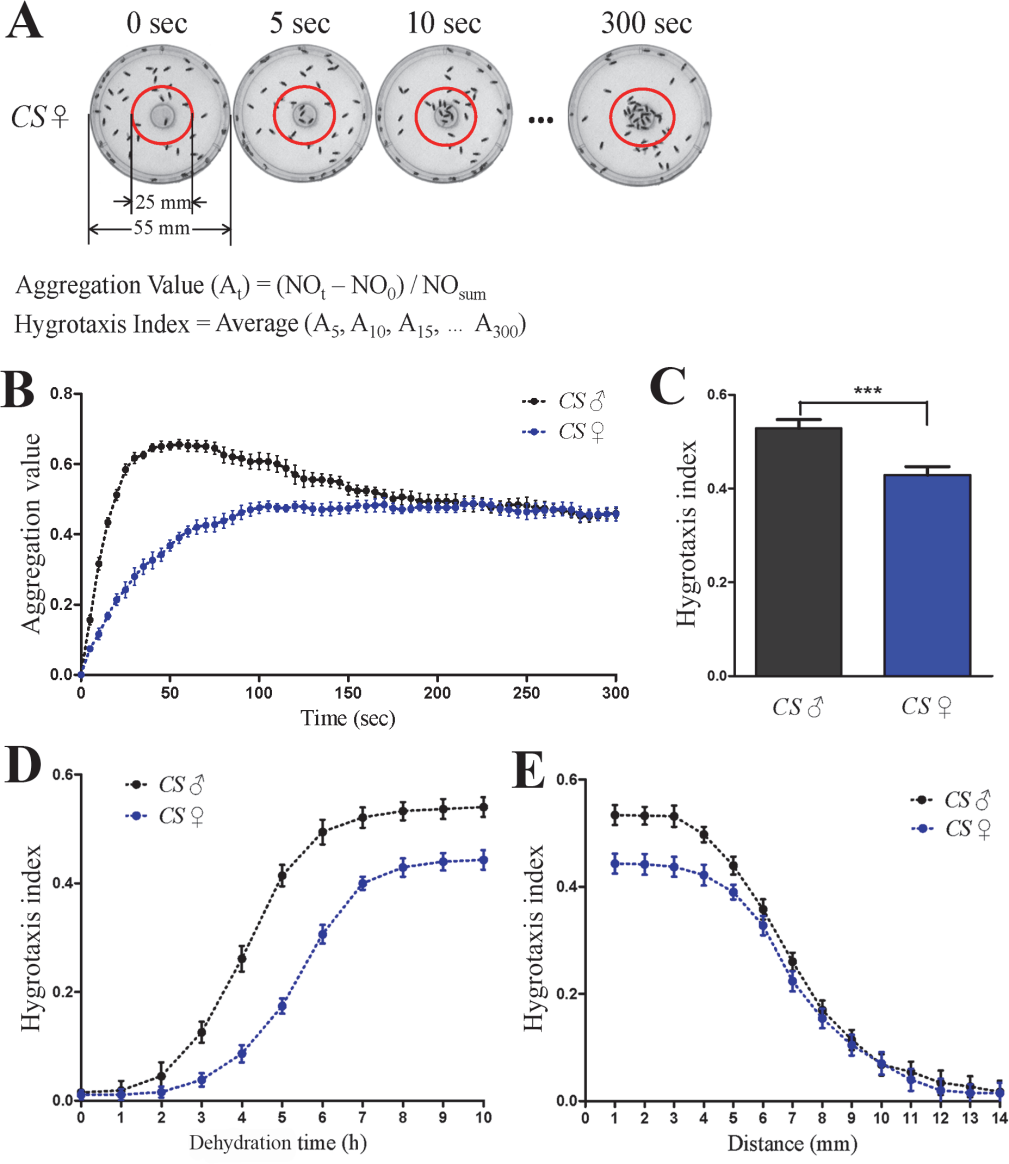
Quantification of hygrotactic behavior in thirsty *Drosophila*. (**A**) Diagram for measuring aggregation value and hygrotaxis index in hygrotactic behavior. Aggregation values represent the aggregation strength at different time points during the test. NO_t_ denotes the number of flies within the red circle at the moment during the test; NO_0_ is the number of flies in the red circle at the beginning of the test; NO_sum_ denotes the total number of flies in the test. Hygrotaxis index is used to represent the strength of hygrotactic behavior. (**B**) Plotting of the aggregation value as a function of time in wild type flies dehydrated for 8 hours. N = 12. (**C**) Hygrotaxis index of wild type flies dehydrated for 8 hours. ***, p < 0.001 (Student’s t test). N = 12. (**D**) The curve of hygrotaxis index vs. dehydration time in wild type flies. N = 12. (**E**) Relationship between hygrotaxis index and the shortest distance from flies to water source. Wild type flies were dehydrated for 10 hours before tests. N = 12. Data are presented as mean ± SEM.

To quantify the hygrotactic behavior in thirsty flies, a circular region was defined in the center of the test dish, and the number of flies in the region was counted every 5 seconds during the test. The aggregation values at different time points were calculated based on the increase of fly numbers in the defined region (Fig. 2A). The curve of the aggregation value reflects the change of aggregation strength during the assay. The curves of the aggregation value of male and female *Canton-S* flies after dehydration for 8 hours are shown in Fig. 2B. To facilitate the comparison of the strength of hygrotactic behavior among different genotypes or under different conditions, we defined the hygrotaxis index as the average of all aggregation values measured at five-second intervals within a five-minute test. For instance, Fig. 2C shows the hygrotaxis index of male and female *Canton-S* flies after eight-hour dehydration.

The hygrotaxis index increased with dehydration time. Fig. 2D shows the hygrotaxis index of *Canton-S* flies for different dehydration times. The males of the *Canton-S* strain dehydrated for over three hours displayed strong hygrotactic behavior, while in the females, dehydration for more than five hours was required to trigger the observed hygrotactic behavior. The longest dehydration time used in our assays was 10 hours, as male flies start to die thereafter. In addition, by adjusting the distance between the water surface and the nylon net on test dish, we tested the influence of the distance between flies and the water on the strength of hygrotactic behavior. The results showed that when the shortest distance between flies and water exceeded 12 mm, even the flies dehydrated longest (10 h dehydration) did not exhibit any visible hygrotactic behavior (Fig. 2E).

### The third antennal segment mediates hygrotactic behavior

**Fig 3:**
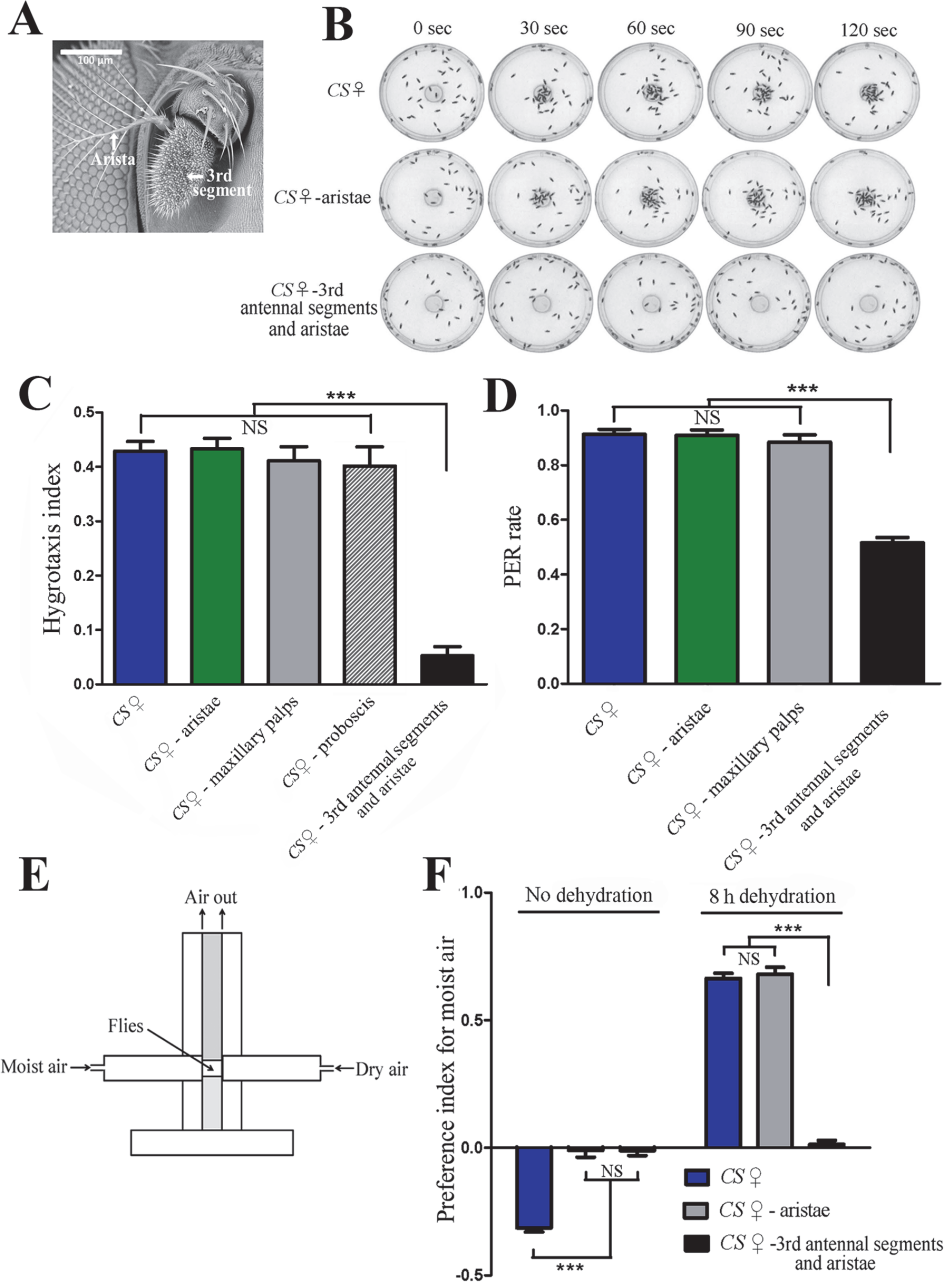
Third antennal segments are required for hygrotactic behavior. (**A**) Scanning electron microscope photograph showing the third antennal segment and arista of *Drosophila*. (**B**) The hygrotactic behavior was tested in wild type flies and the wild type flies that had removed aristae or both third antennal segments and aristae. Ablation of the third antennal segments and aristae abolished hygrotactic behavior, while removing the aristae alone did not affect hygrotactic behavior. All tested flies were dehydrated for 8 hours. (**C**) Ablation of the third antennal segments and aristae greatly reduced the hygrotaxis index in wild type flies dehydrated for 8 hours. N = 12. (**D**) Ablation of the third antennal segments and aristae significantly reduced moisture-induced PER rate in wild type flies dehydrated for 8 hours, while removing the aristae alone did not affect the PER rate. N = 8 trials with 50 flies per trial per condition. (**E**) Schematic diagram of the T-maze apparatus used in the humidity choice assay. Two tubes in the apparatus were filled with moist air (~99% RH) and dry air (~3% RH), respectively. Flies were placed between the two tubes, and allowed to make a choice between the two types of air. (**F**) The effect of sensory organ ablation on humidity choice behavior in wild type flies. Before dehydration, the intact wild type flies avoid moist air, while the wild type flies with the ablation of aristae or both the third antennal segments and aristae showed no bias towards dry or moist air. After dehydration for 8 hours, the intact flies and flies with aristae removed exhibited a strong preference for moist air, while flies with both third antennal segments and aristae removed showed no humidity preference. N = 15. NS, not significant (p > 0.05); ***, p < 0.001 (ANOVA with Tukey post hoc test). Data are presented as mean ± SEM.

Previous studies revealed that in flies, the hygroreceptors were located in the antenna [10–19], and demonstrated that flies with bilateral ablation of aristae or both the third antennal segments and aristae could not distinguish between dry air and moist air [14–15]. Using a similar ablation approach, we next examined whether the antennae were required for hygrotactic behavior in thirsty flies. When the third antennal segments and aristae were removed, flies of the dehydrated wild type strain did not aggregate near water source, suggesting that the flies could not locate the water source without the third antennal segments and aristae (Fig. 3B, C). However, the dehydrated wild type flies with aristae ablated alone exhibited normal hygrotactic behavior, just as observed in intact flies (Fig. 3B, C) suggesting that the third antennal segment, but not arista was required for the attraction of flies to moisture. Examination of the locomotor ability indicated that ablation of the third antennal segments and aristae had no effect on the locomotion of these flies (S3 Fig.). Thus, the observed deficit in hygrotactic behavior in ablated flies was not due to defects in locomotion. Furthermore, ablation of the maxillary palps or proboscis did not affect hygrotactic behavior either (Fig. 3C).

In addition, we tested the effect of sensory organ ablation on the PER rate induced by moisture in thirsty flies. Thirsty flies with ablated aristae displayed a similar PER rate to that observed in intact flies. However, ablation of both the third antennal segments and aristae significantly reduced the PER rate in thirsty flies (Fig. 3D). Interestingly, about half of the flies retained PER ability, even though hygrotactic behavior was nearly abolished in thirsty flies whose third antennal segments and aristae were removed. These results suggested that other sensory organs were involved in detecting moisture besides the hygroreceptors located in the antennae. Furthermore, ablation of maxillary palps did not affect PER behavior in thirsty flies (Fig. 3D).

To confirm that the third antennal segment, rather than the arista, is essential for the attraction to moisture, we performed T-maze humidity choice assays and tested the effect of sensory organ ablation on humidity choice behavior. In contrast to the observed avoidance behavior before dehydration, intact wild type flies were strongly attracted by moist air after dehydration for eight hours, which was consistent with results from a previously published work [8]. Although flies with their aristae removed alone exhibited no bias towards dry or moist air before dehydration, they showed strong preference for moist air after dehydration for eight hours. The flies with both the third antennal segments and aristae removed showed no preference for dry or moist air before or after dehydration (Fig. 3F). Based on the above results, we conclude that aristae are required for avoidance behavior of flies toward moist air in hydration status, while the third antennal segments play an essential role in mediating hygrotactic behavior and water localization in the thirst status.

### Mushroom body α/β surface and posterior neurons are required for hygrosensation

To reveal the neural basis of the hygrotactic behavior, we used a neural silencing screen to identify the neurons that are required for hygrotactic behavior. To block neural activity, tetanus toxin light chain (TNT) [23] that prevented the release of neurotransmitter was expressed in specific neurons in the adult fly brain using Gal4 driver lines [24–25], and the effect of neural silencing on hygrotactic behavior in thirsty flies was examined.

**Fig 4:**
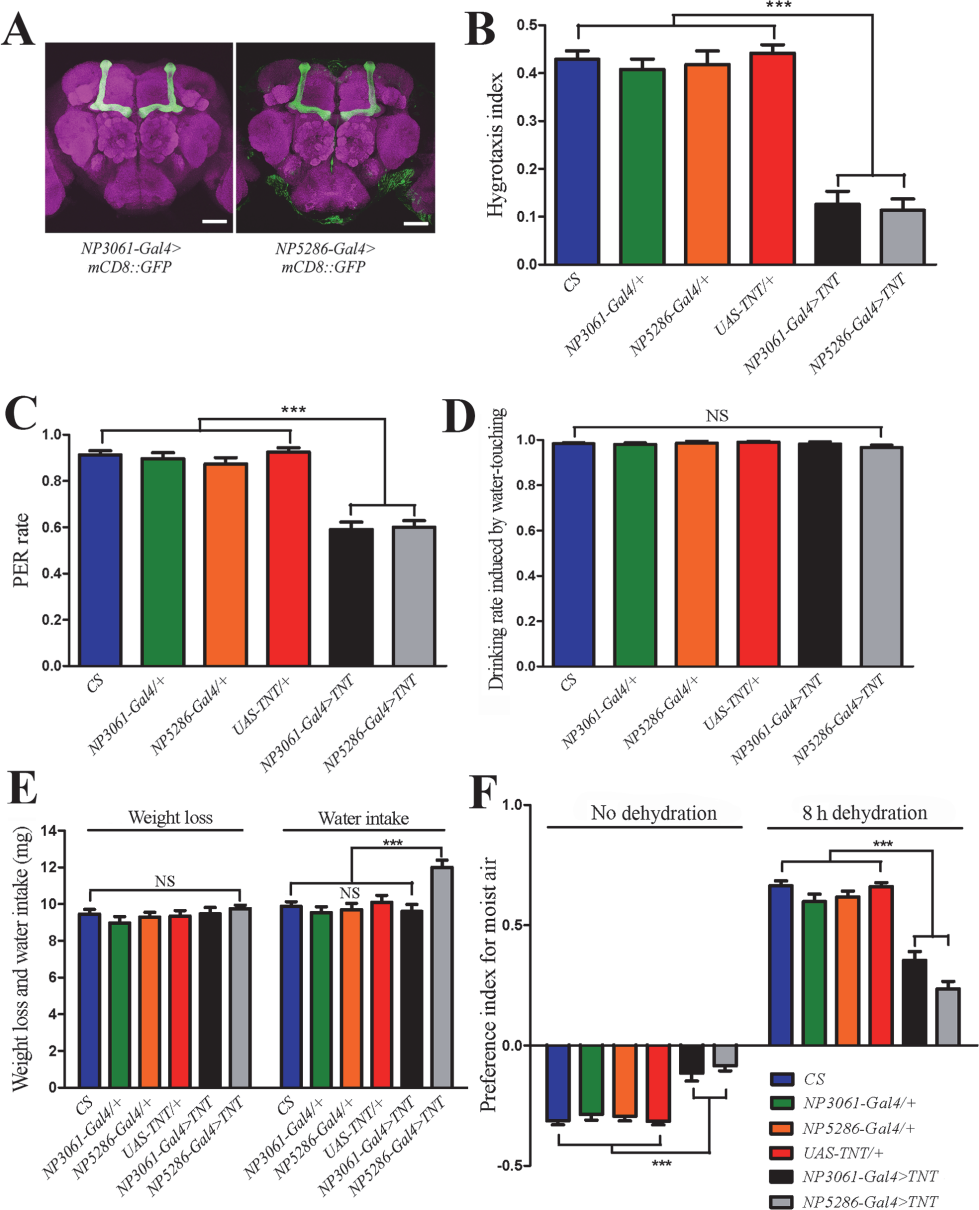
MB α/βsp neurons are required for hygrosensation. (**A**) Immunofluorescent detection of mCD8::GFP driven by *NP3061-Gal4* and *NP5286-Gal4* in the adult fly brain. Both Gal4 lines display specific GFP expression in MB α/βsp neurons, which had been characterized in a previous work [27]. Scale bar, 50 μm. (**B**) Inhibiting synaptic output from MB α/βsp neurons (*NP3061-Gal4 > TNT*, *NP5286-Gal4 > TNT*) impairs the hygrotactic behavior in flies dehydrated for 8 hours. N = 12. (**C**) Inhibiting synaptic output from MB α/βsp neurons significantly reduced the rate of moisture-induced PER in dehydrated flies. N = 8 trials with 50 flies per trial per genotype. (**D**) Blocking the activity of MB α/βsp neurons did not affect the water-drinking behavior of dehydrated flies. N = 8 trials with 50 flies per trial per genotype. (**E**) Blocking the activity of MB α/βsp neurons did not reduce weight loss during 8 hours of dehydration or water intake within 10 minutes of water-feeding following dehydration (flies carrying *NP5286-Gal4 > TNT* drank more water than wild type and control flies). Data represent the weight variations of 50 female flies. N = 12. (**F**) In T-maze humidity choice assays, the flies expressing TNT driven by *NP3061-Gal4* or *NP5286-Gal4* exhibited impaired avoidance behavior toward moist air before dehydration, and also showed reduced preference for moist air after eight-hour dehydration. N = 15. NS, not significant (p > 0.05); ***, p < 0.001 (ANOVA with Tukey post hoc test). Data are presented as mean ± SEM.

We screened about 400 Gal4 driver lines, and identified 32 lines that showed strong and reproducible deficits in hygrotactic behavior when crossed with a *UAS-TNT* transgenic line. Using a *UAS-mCD8*::*GFP* reporter [26], 14 Gal4 driver lines were found to have expression patterns in the mushroom bodies (MB). Among these, two Gal4 driver lines, NP3061 and NP5286, render the most restricted expression in the adult fly brain. They specifically label MB α/β surface and posterior (MB α/βsp) neurons (Fig. 4A) [27]. After dehydration for eight hours, both *NP3061-Gal4* > *TNT* and *NP5286-Gal4* > *TNT* flies exhibited defects in hygrotactic behavior (Fig. 4B). In addition, the PER rate induced by moisture was also significantly reduced in dehydrated flies carrying *NP3061-Gal4* > *TNT* or *NP5286-Gal4* > *TNT* transgenes (Fig. 4C). To exclude the possibility that *NP3061-Gal4* > *TNT* and *NP5286-Gal4* > *TNT* flies have defects in thirst perception which can also cause hygrotactic defects, we tested the water-drinking behavior in *NP3061-Gal4* > *TNT* and *NP5286-Gal4* > *TNT* flies after eight-hour dehydration. Our results showed that these flies exhibited normal drinking behavior, similar to that of wild type and control flies (Fig. 4D). We then measured the weight loss of the tested flies during dehydration, as well as the water intake during water-feeding following dehydration. The results showed that the flies carrying *NP3061-Gal4* > *TNT* or *NP5286-Gal4* > *TNT* did not show less weight loss or water intake than that observed in wild type and control flies (Fig. 4E). Thus, these data further confirmed that the inhibition of MB α/βsp neurons did not impair thirst perception in flies. Moreover, the *NP3061-Gal4* > *TNT* and *NP5286-Gal4* > *TNT* flies exhibited normal locomotor abilities, similar to that of wild type flies (S3 Fig.). MB α/β lobe is composed of surface, posterior and core neurons [27], and we found that the MB α/β core neurons were not required for hygrotactic behavior because the flies expressing TNT driven by *17d-Gal4* that specifically labels MB α/β core neurons [28] showed normal hygrotactic behavior, just as wild type flies did (data not shown).

The olfactory system in *Drosophila* exhibits a well-known pathway that connects the third antennal segment to the MB [27, 29]. We therefore tested whether the olfactory neural circuit played a role in transmitting humidity stimuli to the MB. In the olfactory neural circuit, olfactory information is sent via the olfactory sensory neurons (OSNs) to the antennal lobe, and then via projection neurons (PNs) to the MB [29]. *Orco-Gal4* labels most of OSNs [30], while *GH146-Gal4* is used extensively for labeling the PNs [31–33]. We found that blocking most of the OSNs or PNs by expressing TNT under the control of *Orco-Gal4* or *GH146-Gal4* did not affect the hygrotactic behavior in thirsty flies (S4 Fig.). In addition, the deletion of ORCO, the odorant co-receptor required for functioning of most OSNs [29, 34] in flies, did not cause any detectable defects in hygrotactic behavior (S4 Fig.). These results implied that the olfactory system may not participate in moisture sensation in hygrotactic behavior.

Furthermore, we used the T-maze humidity choice assay to test the effect of silencing MB α/βsp neurons on humidity choice behavior in hydrated and thirsty flies. We found that expression of TNT in MB α/βsp neurons driven by *NP3061-Gal4* or *NP5286-Gal4* not only impaired avoidance behavior toward moist air in hydrated flies, but also affected the preference for moist air in thirsty flies (Fig. 4F).

The above results indicate that MB α/βsp neurons play an important role in hygrosensory circuits, and that the synaptic output from these neurons is required for different behavioral responses induced by humidity.

## Discussion

Humidity is important for the survival of animals because it affects the water maintenance and temperature regulation of animal bodies. Most terrestrial animals can detect the variation of humidity in their environment, and exhibit preferences for specific levels of humidity [8, 14]. In our study of behavioral responses to moisture in thirsty *Drosophila*, we demonstrate that hygrosensation is not only useful for identifying a suitable living environment, but also plays an important role in detection and localization of water sources in thirsty insects. Further studies are needed to investigate whether hygrosensation has a similar role in other animals such as reptiles, birds and mammals.

So far, the molecular and cellular basis for hygrosensation is unclear, however, our findings provide evidence that two sets of molecules and cells function in the moisture-response behaviors of *Drosophila*. When allowed to choose between dry air (~3% RH) and moist air (~99% RH), normal wild type flies prefer dry air over moist air [14–15]. However, humidity preference in wild type flies is adaptive and changes according to the status of body osmolality. Perttunen and Erkkila reported that when flies were dehydrated for several hours, they showed a preference for the moist air in their humidity choice assay [8]. Furthermore, the results of our assay demonstrated that thirsty flies are able to detect and locate water sources through sensing the gradient of moisture in their immediate environment. Here we reveal that arista and the third antennal segment play different roles in mediating moisture-induced behaviors, with our data suggesting that there exist two types of hygroreceptors in *Drosophila*: the hygroreceptors in the arista mediate moisture-aversive behavior in hydrated flies, while the hygroreceptors in the third antennal segment play an essential role in hygrotactic behavior in thirsty flies. Additionally, Liu et al. reported that two channels from TRP family—Nan and WTRW—were necessary for normal flies to avoid moist air in their T-maze humidity choice assay [15]. However, we found that *nan* mutant and *wtrw* neural knockdown flies still exhibited strong hygrotactic behavior, comparable to that of wild type flies (S5 Fig.), suggesting that two sets of molecules function in the moisture-response behaviors of *Drosophila*.

In animals, higher brain centers are essential to integrate multiple inputs from sensory neurons and elicit adaptive behavioral responses. In *Drosophila*, mushroom body is a brain center that receives inputs from multiple modalities of sensory information, including olfactory inputs from the antennal lobe as well as putative gustatory inputs from the subesophageal ganglion (SOG), and modulates behavioral output [27, 35–38]. MB α/β neurons were shown to receive olfactory input from the antennal lobe [27, 38], and are required for the memory retrieval in olfactory associative learning [39–40]. Here, we demonstrated that MB α/βsp neurons were required for both moisture-attractive behavior in thirsty flies and moisture-aversive behavior in hydrated flies. One reasonable explanation is that the α/β lobe of the MB is the neural center that receives and processes sensory input of humidity stimuli, and that the output from this lobe is necessary for eliciting humidity-related behaviors. Further investigation is needed to dissect the precise neural circuits involved in the transmission of humidity stimuli to the MB neurons. Moreover, it remains an interesting question as to how MB α/β lobe neurons integrate information from the hygrosensory pathways and switch between two distinct behavioral patterns according to the status of body osmolality.

In conclusion, our results presented here reveal the duality of both function and structure in the hygrosensation in *Drosophila*. The mutation screen and new neural silencing screen in both hygrotaxis assay and humidity choice assay are underway to reveal the molecular and cellular mechanisms of this important, yet rarely investigated, sensory system.

## Supporting Information

S1 Fig The hygrotactic behavior was triggered by dehydration rather than starvation. The time of dehydration was 8 hours; the time of starvation was 10 hours.
(TIF)

S2 Fig Volatile compounds other than water (Ethanol, acetic acid and ethyl acetate) failed to induce aggregation behavior in thirsty flies. All flies were dehydrated for 8 hours.
(TIF)

S3 Fig Ablation of the third antennal segments or silencing of MB α/βsp neurons did not impair locomotor ability. Data show locomotion distances of flies within 10 seconds. All distances were larger than the radius (27.5 mm) of the dish used in the hygrotaxis assay, therefore 10 seconds were deemed sufficient for all tested flies to migrate from the edge to the center of the dish during hygrotactic tests. NS, not significant (p > 0.05). N = 36. Data are presented as mean ± SEM.
(TIFF)

S4 Fig Olfactory system may not function in moisture sensation in hygrotactic behavior. Neither deleting odorant co-receptor ORCO nor blocking most of the ORNs (*Orco-Gal4 > TNT*) or PNs (*GH146-Gal4 > TNT*) affected the hygrotactic behavior in flies dehydrated for 8 hours. NS, not significant (p > 0.05). N = 12. Data are presented as mean ± SEM.
(TIF)

S5 Fig Thirsty flies with *nan* mutation or *wtrw* neural knockdown exhibited normal hygrotactic behavior. All flies were dehydrated for 8 hours. NS, not significant (p > 0.05). N = 12. Data are presented as mean ± SEM.
(TIFF)
