# Supplementary figures and images for: A Novel Assay Reveals Hygrotactic Behavior in Drosophila
Source: PLoS One. 2015 Mar 4;10(3):e0119162. doi: 10.1371/journal.pone.0119162 (PMC4349581; doi:10.1371/journal.pone.0119162)

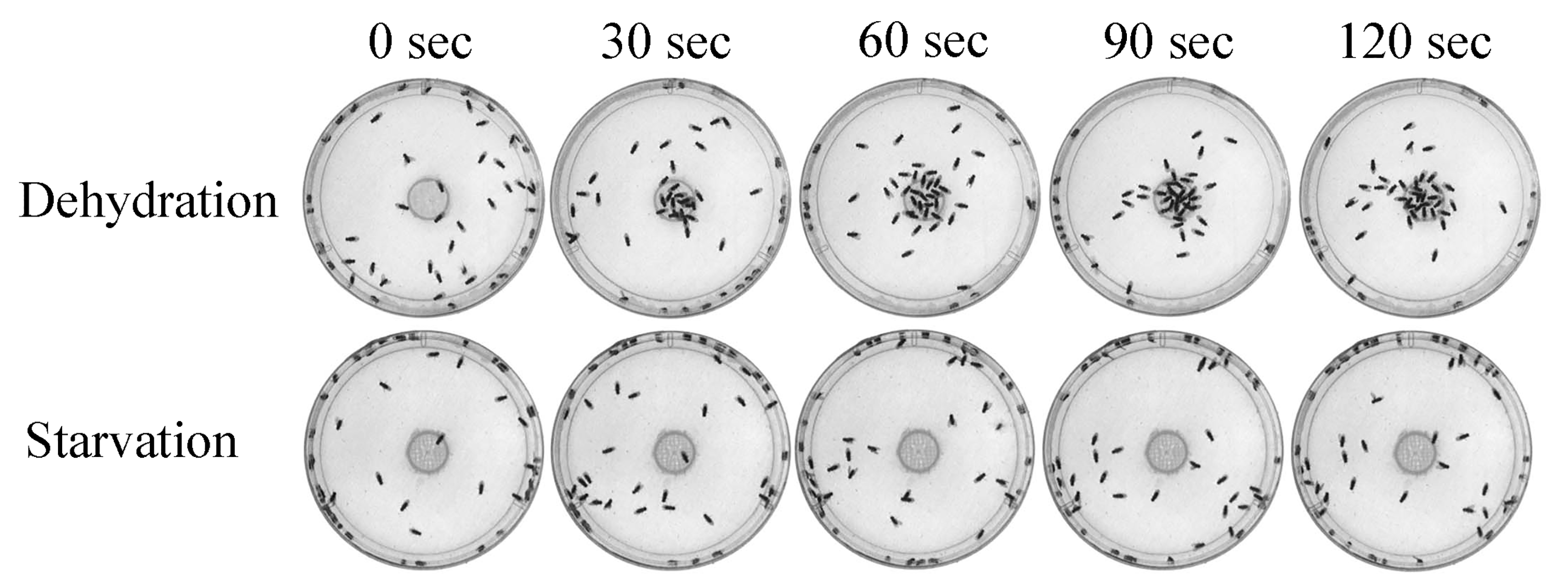

Supplement: S1 Fig — The time of dehydration was 8 hours; the time of starvation was 10 hours. (TIF) [file pone.0119162.s001.tif]

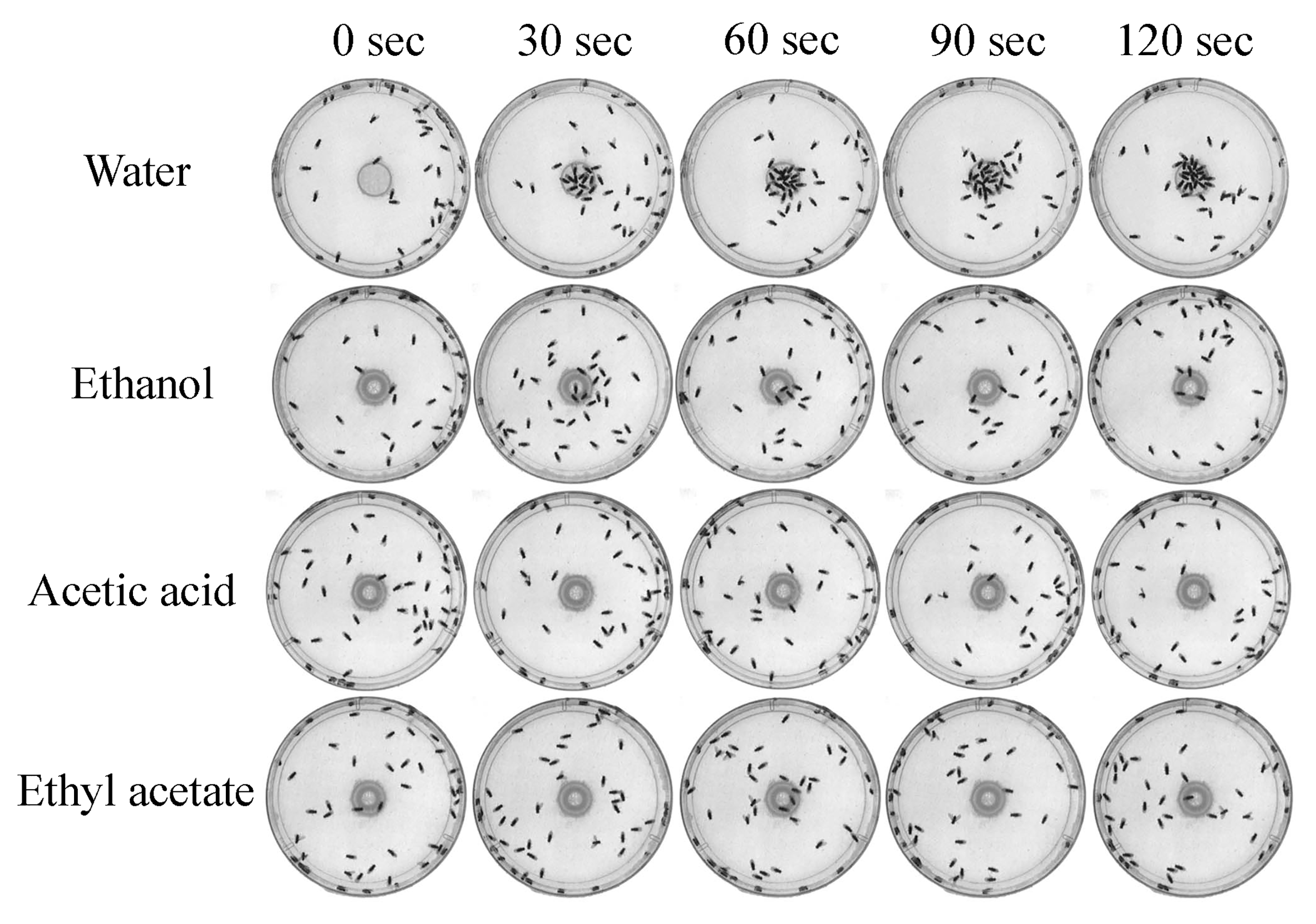

Supplement: S2 Fig — All flies were dehydrated for 8 hours. (TIF) [file pone.0119162.s002.tif]

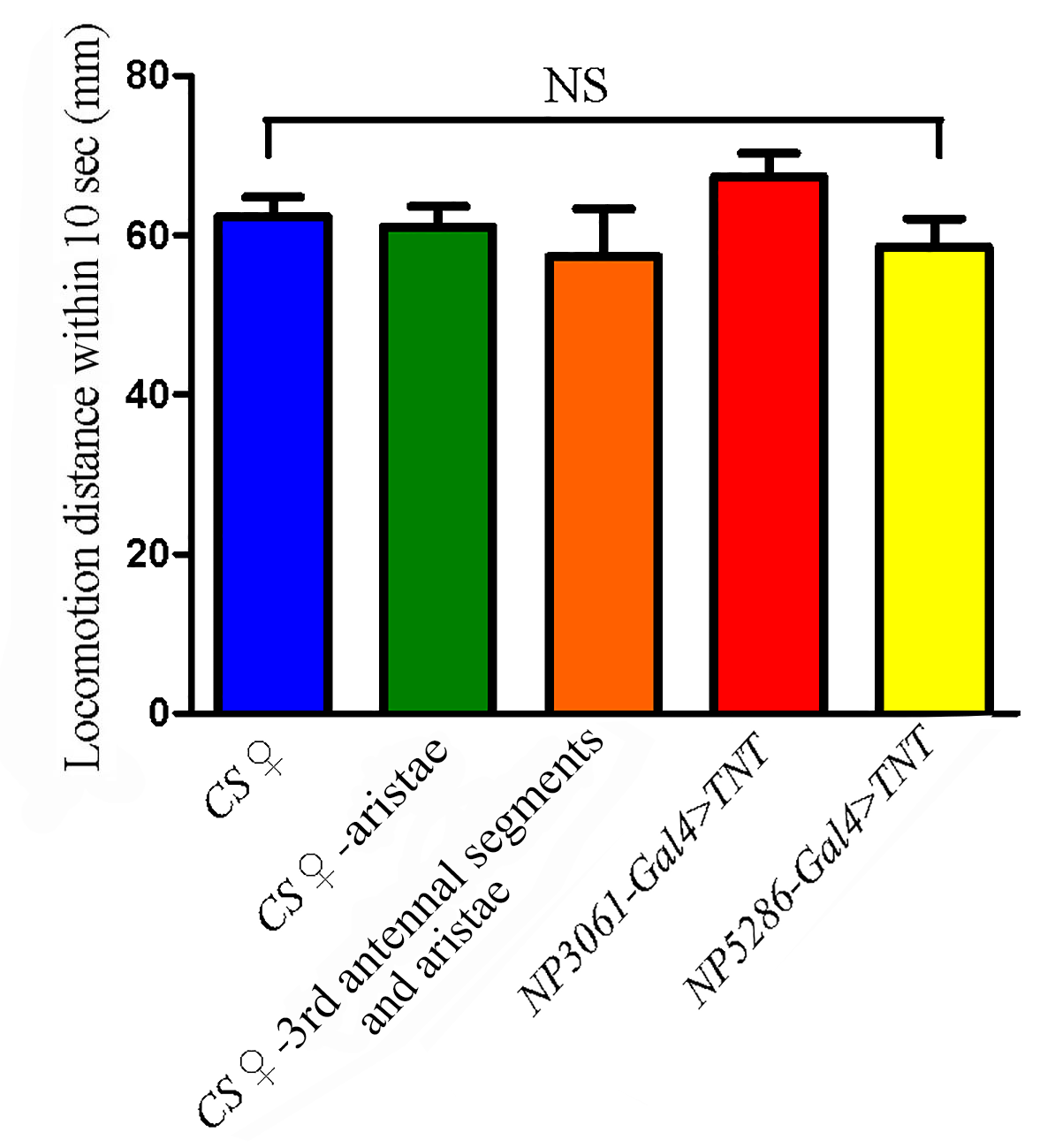

Supplement: S3 Fig — Data show locomotion distances of flies within 10 seconds. All distances were larger than the radius (27.5 mm) of the dish used in the hygrotaxis assay, therefore 10 seconds were deemed sufficient for all tested flies to migrate from the edge to the center of the dish during hygrotactic tests. NS, not significant (p > 0.05). N = 36. Data are presented as mean ± SEM. (TIFF) [file pone.0119162.s003.tiff]

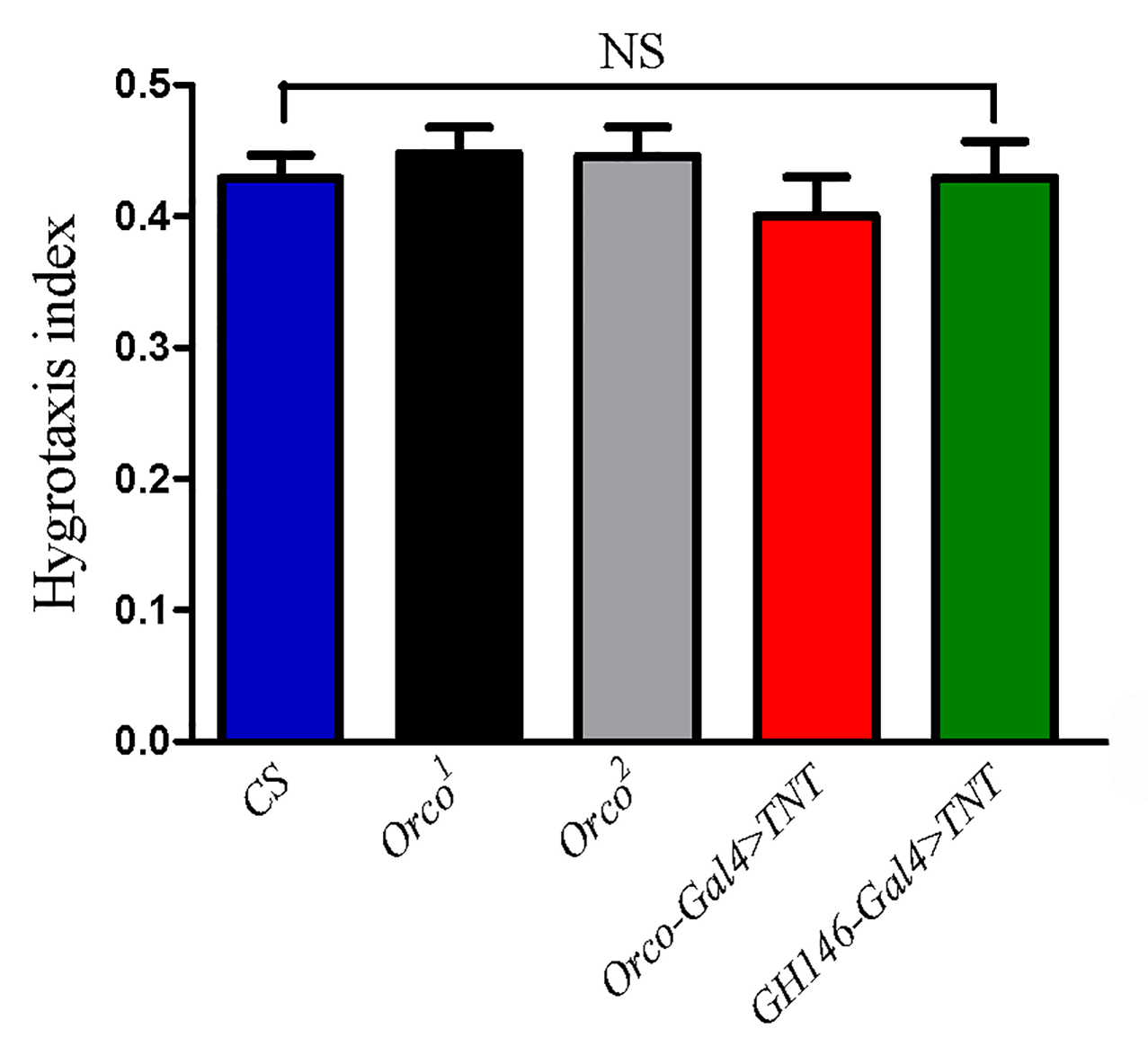

Supplement: S4 Fig — Neither deleting odorant co-receptor ORCO nor blocking most of the ORNs (Orco-Gal4 > TNT) or PNs (GH146-Gal4 > TNT) affected the hygrotactic behavior in flies dehydrated for 8 hours. NS, not significant (p > 0.05). N = 12. Data are presented as mean ± SEM. (TIF) [file pone.0119162.s004.tif]

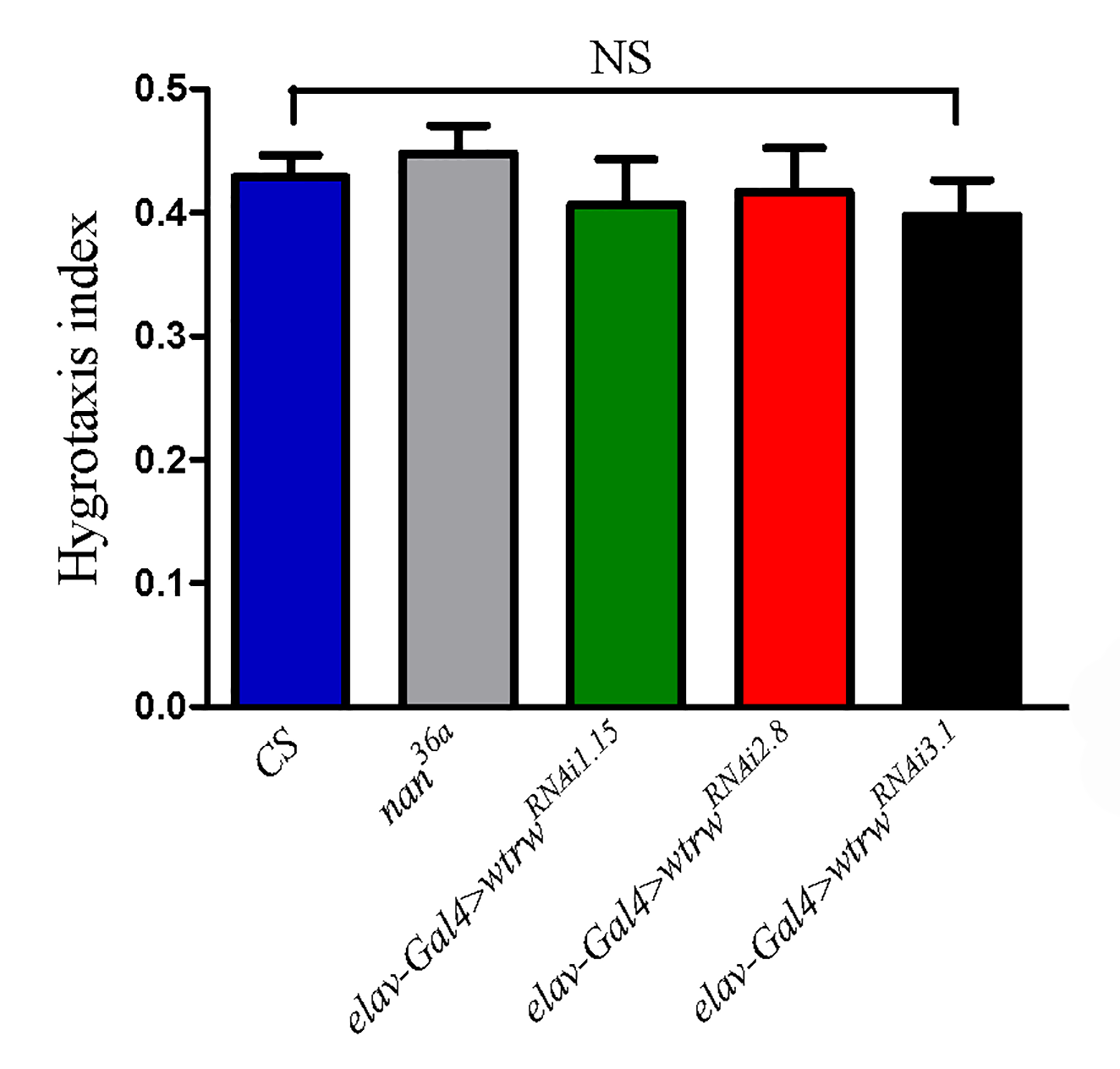

Supplement: S5 Fig — All flies were dehydrated for 8 hours. NS, not significant (p > 0.05). N = 12. Data are presented as mean ± SEM. (TIFF) [file pone.0119162.s005.tiff]
